# Radiotherapy in the Era of Immunotherapy With a Focus on Non-Small-Cell Lung Cancer: Time to Revisit Ancient Dogmas?

**DOI:** 10.3389/fonc.2021.662236

**Published:** 2021-04-21

**Authors:** Jonathan Khalifa, Julien Mazieres, Carlos Gomez-Roca, Maha Ayyoub, Elizabeth Cohen-Jonathan Moyal

**Affiliations:** ^1^Department of Radiotherapy, Institut Claudius Regaud/Institut Universitaire du Cancer de Toulouse – Oncopole, Toulouse, France; ^2^Institut National de la Santé et de la Recherche Médicale U1037, Centre de Recherche contre le Cancer de Toulouse, Toulouse, France; ^3^Department of Pulmonology, Centre Hospitalo-Universitaire Larrey, Toulouse, France; ^4^Université Toulouse III Paul Sabatier, Toulouse, France; ^5^Department of Medical Oncology, Institut Claudius Regaud/Institut Universitaire du Cancer de Toulouse – Oncopole, Toulouse, France

**Keywords:** radiotherapy, immunotherapy, immune check point inhibitors (ICI), abscopal effect, lymphopenia, non-small-cell lung cancer (NSCLC), adscopal effect

## Abstract

Radiation-induced immune effects have been extensively deciphered over the last few years, leading to the concept of the dual immune effect of radiotherapy with both immunostimulatory and immunosuppressive effects. This explains why radiotherapy alone is not able to drive a strong anti-tumor immune response in most cases, hence underlining the rationale for combining both radiotherapy and immunotherapy. This association has generated considerable interest and hundreds of trials are currently ongoing to assess such an association in oncology. However, while some trials have provided unprecedented results or shown much promise, many hopes have been dashed. Questions remain, therefore, as to how to optimize the combination of these treatment modalities. This narrative review aims at revisiting the old, well-established concepts of radiotherapy relating to dose, fractionation, target volumes and organs at risk in the era of immunotherapy. We then propose potential innovative approaches to be further assessed when considering a radio-immunotherapy association, especially in the field of non-small-cell lung cancer (NSCLC). We finally propose a framework to optimize the association, with pragmatic approaches depending on the stage of the disease.

## Introduction

For more than a century, radiotherapy (RT) has been the cornerstone for the treatment of cancer. The classical radiobiological mechanisms underlying tumor cell death are well known, mainly involving deoxyribonucleic acid chain (DNA) damage, either directly or *via* water radiolysis and the production of free radicals and reactive oxygen species (ROS). The relative biologic effectiveness of radiation is influenced by several mechanisms known as the ‘5Rs’: repair of sublethal damage, repopulation, redistribution within the cell cycle, reoxygenation and intrinsic radiosensitivity, which mostly explains variations in radiosensitity/radioresistance for a given tissue/tumor (1).

Conventional RT consists in delivering once daily fractions of 1.8-2.2 Gy for 5-8 weeks, as this empirical approach turned out to achieve a differential effect between tumor cells and normal tissue. With the advances in dose delivery, patient immobilization and repositioning and tumor motion management, stereotactic RT has emerged, enabling the delivery of higher biological effective doses (BED) in fewer fractions and with a sharp dose fall-off.

Immunotherapy (IO) to restore and/or to boost anti-tumor immunity, especially with immune check-point inhibitors (ICI), has changed the standard of care in many fields of oncology for a decade. For example, the PACIFIC trial led to an unprecedented gain in progression-free survival (PFS) and overall survival (OS) for the management of non-resectable stage III non-small-cell lung cancer by adding durvalumab as maintenance therapy following chemoradiotherapy (2).

Along with the advances in anti-tumor immunity research, the deciphering of radiation-induced immune effects has led to the concept of a dual immune effect of RT with both immunostimulatory and immunosuppressive effects (3). Briefly, RT can release tumor-antigens (TA) along with the translocation of calreticulin to the tumor cell membranes, leading to tumor cell phagocytosis (4) and the activation of the cytosolic DNA sensing cGAS/STING pathway, with in turn induction of interferon β (IFN-β) (5), and the release of damage-associated molecular patterns (DAMPs) (such as heat shock proteins, high mobility group box 1 molecules (HMGB1) or adenosine triphosphate (ATP)). These DAMPs are recognized by toll-like receptors (TLRs) expressed at the surface of dendritic cells (DCs), and can promote processing and cross-presentation of TA by IFN-β-induced mature DCs (6). Following this immunogenic cell death, DCs then migrate to the tumor-draining lymph nodes and prime CD8^+^ T cells (7), with in turn leukocyte extravasation and recruitment to the tumor site through chemokine secretion by tumor cells and other cell types in the tumor micro-environment (CXC motif chemokine ligand (CXCL)9, CXCL10, and CXCL16) (8, 9). Once T cells have infiltrated the tumor tissue, they encounter tumor cells with the radiation-induced expression of several surface molecules and receptors, such as MHC-I molecules (10), the TNF-R superfamily (11, 12) and ligands for the NKG2D receptor (13), leading to enhanced tumor cell killing by CD8^+^ T cells and NK cells.

Together with this radiation-induced *in situ* “vaccination”, RT can induce immunosuppressive effects *via* several mechanisms: upregulation of PD-L1 levels on tumor cells *via* IFN-γ released by CD8^+^ T cells and of PD-1 levels on CD8^+^ tumor infiltrating lymphocytes (TILs), contributing to T cell exhaustion (14, 15); direct depletion of circulating lymphocytes and lymphoid progenitors in primary and secondary lymphoid organs (16, 17); enhancement of immune suppressive pathways (mostly: HIF1α upregulation, increased colony-stimulating factor 1 (CSF1) levels, induction of TGF-β and generation of adenosine from ATP), which in turn lead to a suppressive tumor micro-environment (TME) with induction of CD4^+^CD25^+^ regulatory T cells (T-reg) proliferation, M2 polarization of tumor-associated macrophages (TAMs), and myeloid-derived suppressor cells (MDSC) activation (18).

Overall, this dual effect can explain why RT alone is not able to drive a strong anti-tumor immune response with a so-called “abscopal” effect in most cases and underlies the rationale for combining RT with IO, not only to amplify the *in situ* vaccination effect but also to overrule immunosuppressive effects. This rationale has generated considerable interest in this field, and around 700 trials are currently ongoing assessing different regimens of such associations in oncology. However, while some trials have provided unprecedented results and shown much promise (2, 19–21), others have led to disappointment (22–24). These discrepancies leave many open questions regarding the optimal combinations of these treatment modalities.

This narrative review aims at revisiting the old, well-established concepts of RT relating to dose, fractionation, target volumes and organs at risk in the era of IO, in order to propose potential innovative approaches to be further assessed when considering an RT + IO association, especially in the field of non-small-cell lung cancer (NSCLC).

Searches for original and review articles in the PubMed and Google Scholar databases were conducted until September 2020. General search terms (including both Medical Subject Headings (MeSH) and free text words) included the following: “radiotherapy”, “immunotherapy”, “immune checkpoint inhibitor”, “anti-PD(L)1”, “abscopal effect”, “lung cancer”, “non-small-cell lung cancer”, “lymphopenia”. Individual bibliographies were reviewed for additional relevant references.

## Which RT + IO Association for Which Objective?

**Figure 1:**
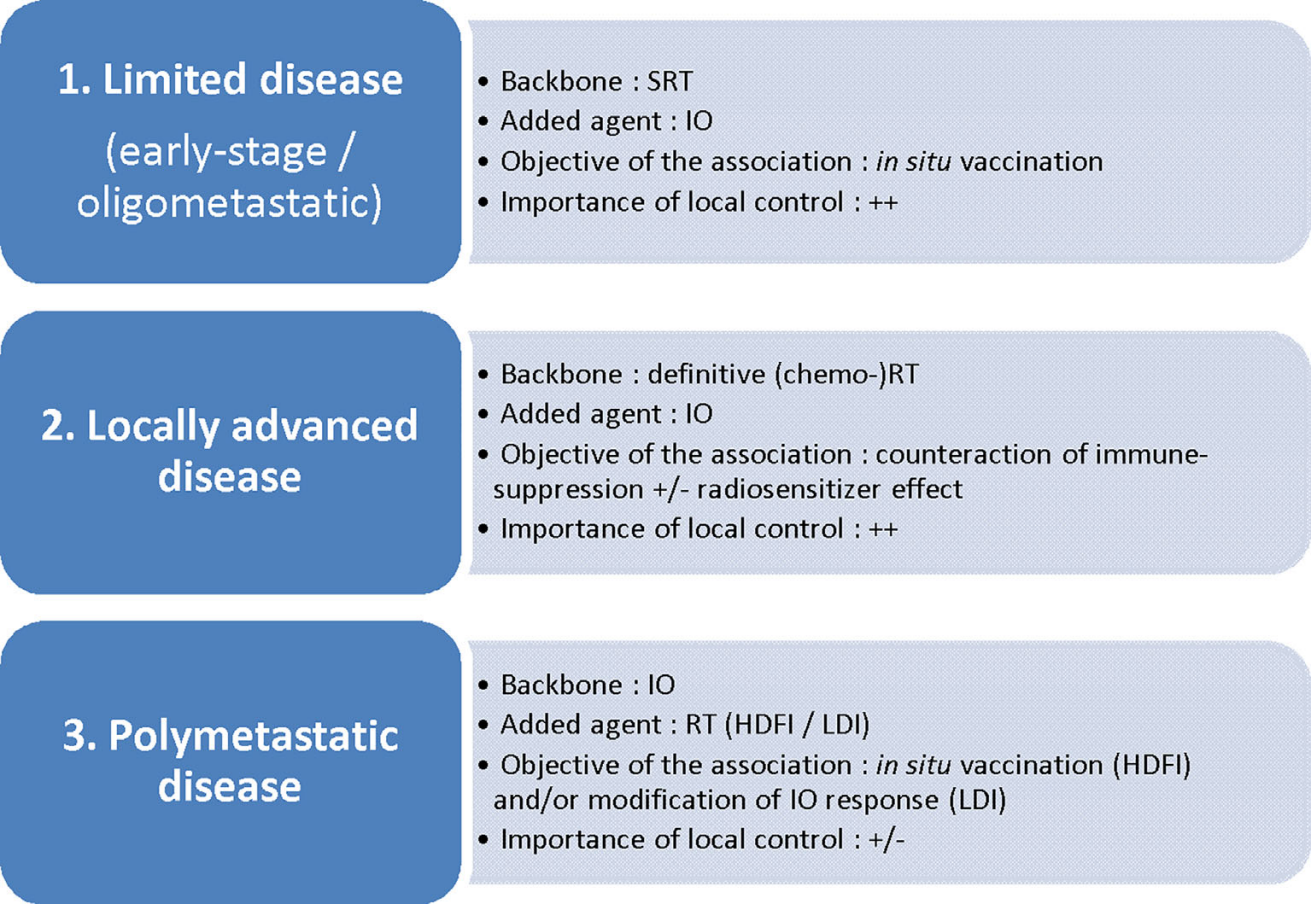
Disease setting and radiotherapy/immunotherapy combinations: which association for which objective? SRT, stereotactic radiotherapy; IO, immunotherapy; (chemo-)RT, (chemo-)radiotherapy; HDFI, high-dose per fraction irradiation; LDI, low-dose irradiation.

To establish the best RT scheme in the context of an RT + IO association, one should first define the main objective of such an approach (25) (**Figure 1**).

Schematically, the first objective is to promote the *in situ* vaccination effect of RT, either by adding IO to a short course of ablative (i.e. tumoricidal) RT towards the whole tumor sites (in early-stage disease or oligometastatic disease; in this case, IO in itsef also addresses the micrometastatic disease), or by adding RT at one or several metastatic sites to IO (polymetastatic disease). Several IO agents potentially trigger such an effect: activation of DCs *via* TLR agonists (26) or CD40 agonists (27); enhancement of T-cell priming *via* CTLA-4 antagonists (28, 29), OX40 agonists (30) or PD-1/PD-L1 antagonists (as PD-1 acts by inhibiting signaling downstream of the CD28 costimulatory receptor following B7-ligation) (31); enhancement of killing by effector T cells, mostly *via* PD-1/PD-L1 antagonists (14, 15).

Another approach consists in counteracting immunosuppressive signals induced by conventional daily definitive (chemo-)radiotherapy schedules for locally advanced disease, due to the enhancement of immunosuppressive pathways as described above. In this case, IO and especially ICI can be used preferentially as a consolidative agent immediately following standard of care (chemo-)radiotherapy, then addressing the micrometastatic disease. Indeed, the restoration of the effective functions of TILs with ICI given concomitantly with tumor irradiation can be counterproductive owing to the profound suppression of TILs induced by daily RT. This is the rationale underpinning the PACIFIC trial, in which the addition of durvalumab following chemo-radiotherapy for stage III NSCLC led to the reduction of distant metastases and improved PFS and OS (2). The question as to whether IO can also act as a local radiosensitizer through a synergistic effect in this setting remains a matter of debate.

Finally, irradiation may serve as a strategy to modify the response to IO, in order to increase the immunogenicity of “cold tumors” through the homing of TILs or the reprogramming of the TME, inducing macrophage M1 polarization, for example (32).

## Controversies About Dose and Fractionation

Regarding the *in situ* vaccination effect, high dose per fraction irradiation (HDFI), usually through stereotactic radiotherapy (SRT), that delivers a few fractions with a high dose of radiation per fraction (generally above 6-8 Gy) is usually preferred, either as a tumoricidal schedule (e.g. 5 x 8 Gy) or as a non-tumoricidal schedule (e.g. 3 x 6 Gy). It has been shown that the release of intra-cellular peptides following single-fraction radiation took place in a dose-dependent manner *via* three main mechanisms with early and late effects: an increase in old protein degradation, upregulation of defined proteins through the response repair, and an increase in protein synthesis through the mTOR pathway activation. As peptides are the limiting factor, the increased intra-cellular peptide pool led to a dose-dependent increase in MHC class I presentation (10). Besides, Golden et al. showed that each component of immune-cell death following single-fraction radiation (calreticulin cell surface exposure, release of high mobility group box 1 (HMGB1) protein and release of ATP) was also induced in a dose-dependent manner from 2 to 20 Gy (33). Finally, Morisada et al. suggested a dose-dependent effect of radiation on both TA release and T-cell priming, with 8 Gy in a single fraction enhancing these pathways compared to 2 Gy in a single fraction, resulting in increased tumor cell susceptibility to T-cell-mediated killing (34). Importantly, Dewan et al. showed that a 3 x 8 Gy regimen was superior to 5 x 6 Gy in the induction of the abscopal effect and of tumor-specific T-cells, suggesting that this dose-dependent pro-immunogenic effect refers to the dose per fraction more than the total dose (35). However, the same group showed that HDFI (3 x 8 Gy 5 x 6 Gy), but not “ultra”-high single-dose RT (20 Gy x 1), was able to induce an abscopal effect when combined with anti-CTLA-4 (35). Vanpouille-Box et al. showed that the DNA exonuclease Trex1 is induced by radiation doses above a threshold ranging from 12-18 Gy (36). During phagocytosis by myeloid cells, DNA fragments hidden in irradiated tumor cells are released from tumor-derived exosomes to the cytoplasm of myeloid cells (37), and cytosolic DNA stimulates the secretion of IFN-β through the activation of the DNA sensor cGAS and its downstream effector STING, in turn promoting the cross-priming of CD8^+^ T cells (5). Above the threshold for Trex1 activation, DNA fragments are cleared from the cytosol, then precluding the secretion of IFN-β and T cell priming (36). Finally, while classical approaches tend to favor doses per fraction that are as high as possible in the context of SRT, these data suggest that the best SRT schedule for maximizing *in situ* vaccination in combination with IO is the delivery of 8-10 Gy fractions. In the context of early-stage NSCLC, several trials are currently assessing the benefit of IO in addition to standard of care SRT (NCT03110978; NCT03446547; NCT 03050554; NCT03383302). Some of them have implemented doses per fraction of around 10-12 Gy while another approach consists in a traditional fractionation of 3 x 18 Gy with addition of IO acting more as an adjuvant treatment than as a synergistic association to decrease the risk of regional and distant failures following SRT for high-risk stage I disease. In the context of NSCLC oligometastatic disease (generally fewer than five metastases), where SRT to all targets is now classically proposed as an ablative treatment (38, 39), the benefit of IO adjunction to SRT is being assessed in several trials with dose per fraction around 6-10 Gy (NCT03275597). In this perspective, when SRT to brain oligometastases is proposed in a context of IO, a hypofractionated schedule (e.g. 3 fractions of 8-10 Gy) could be better than a classical single fraction of 16-20 Gy. Such a schedule is being tested in patients with recurrent glioblastoma, in association with durvalumab (40). A provocative question is whether tumoricidal irradiation is absolutely required for localized disease when HDFI and IO are combined. This could pave the way for dose de-escalation with the definition of new therapeutic windows exploiting the synergy between RT and IO while decreasing radiation-induced toxicity. Finally, in the context of polymetastatic NSCLC disease, the benefit of the *in situ* vaccination effect of HDFI (tumoricidal or not) to one or several targets using doses per fraction of 6-10 Gy in addition to standard of care IO has been suggested (19–21) and is being assessed in the phase III NIRVANA-Lung trial NCT03774732.

When ICIs have been assessed as consolidation agents following standard of care definitive (chemo-)radiotherapy for locally advanced disease, RT has been delivered mostly in a conventional dose-fractionation schedule (1.8-2 Gy per fraction, one fraction per day, five days per week, to a total of 60-66 Gy for NSCLC) (2, 41, 42). The rationale behind this schedule is based on the linear quadratic model, whereby the optimal dose-fractionation regimen in order to kill cancer cells while sparing surrounding normal tissues may be established. However, the linear quadratic model accounts only for radiation cell killing and does not take the role of the immune system in antitumor responses into account (1). Therefore, the optimization of dose-fractionation chemoradiotherapy regimens for locally advanced disease in the context of IO combination requires careful consideration, as conventional fractionated regimens have been associated with lymphopenia and immune suppression in several types of cancers (16, 17). In this perspective, moderately hypofractionated (2.5-4 Gy per fraction) schedules could be of interest because the acceleration of treatment allowed by hypofractionated schedules could reduce the amount of blood passing through the beam and thus the duration and the severity of radiation-induced T-cell suppression and lymphopenia (43, 44). Indeed, in their study of 115 patients with unserectable stage III NSCLC treated by definitive RT, Zhao et al. found that overall treatment time within 4 weeks was significantly associated with a decreased risk of developing severe lymphopenia in multivariate analysis (44). Notably, in this setting of locally advanced disease, the question whether the addition of IO to (chemo-)radiotherapy can act as a radiosensitizer through a synergistic effect remains open for two main reasons. First, the majority of data regarding *in situ* vaccination have been obtained using a high radiation dose per fraction, corroborating the fact that the pro-immunogenic effects of radiation probably occur in a dose per fraction-dependent manner, provided that the 10-12 Gy threshold is not surpassed (10, 33, 34). Yet, the large fields required in the treatment of locally-advanced disease generally preclude the use of doses per fraction higher than 4 Gy. Second, data regarding the synergistic effects of moderately hypofractionated RT in association with IO are not consistent: while several preclinical studies suggest a benefit of hypofractionated over conventionally fractionated regimens, due to better CD8^+^ T cell dependent primary and abscopal tumor control (45) and reduced recruitment of MDSCs into tumors through the downregulation of vascular endothelial growth factor (VEGF), a recent clinical series of 47 metastatic melanoma patients treated with ipilimumab and RT showed that fraction size ≤ 3 Gy vs > 3 Gy was associated with an improved rate of index lesion response outside the radiation field after adjusting for total radiation dose, site irradiated, timing of ipilimumab, and time from diagnosis to radiation treatment (46). Overall, hypothesizing a potential synergy of moderate hypofractionated RT in combination with IO, and considering the concern of early data of the toxicity of such an association (47) as well as the inconsistent data regarding the dose response effect following chemoradiotherapy in locally advanced NSCLC (48–50), dose de-escalated hypofractionated RT in combination with IO (and especially durvalumab consolidation) in stage III NSCLC is probably an approach to be investigated.

Finally, when considering RT as a response modifier of IO, low-dose irradiation (LDI) with one or a few fractions of 0.5 to 2 Gy has been shown to potentially increase the immunogenicity of “cold tumors” through several mechanisms: preferential induction of T-reg apoptosis compared with effector T cell cells (40); skewing macrophages from an M2 phenotype (promoting tumor growth) towards an inducible nitric oxide synthase-positive (iNOS+) M1 phenotype. These M1 macrophages in turn produce a range of chemokines which facilitate T-cell recruitment and normalize tumor vasculature, inducing T-cell tumor-infiltration (32). Furthermore, an original approach has been proposed combining both HDFI and LDI in the context of the RT + IO association in order to generate *in situ* vaccination together with T-cell homing towards tumor sites (25). This hypothesis was corroborated in a preclinical study with bilateral mouse tumor models in which the authors suggested that HDFI of the primary tumor combined with LDI of the abscopal tumor and anti-PD-1 therapy achieved the best abscopal response, compared to HDFI + anti-PD-1, HDFI + LDI or LDI + anti-PD-1. The enhanced abscopal response was correlated with increased infiltration of CD8^+^ effector T cells and upregulated expression of T-cell attracting chemokines (51). Clinical evidence of such LDI in association with HDFI has also been suggested in several reports. In a post-hoc analysis of three immunoradiation trials monitoring SRT with HDFI to a limited number of targets in association with IO, the out-of-field response of non-target lesions among 26 patients was statistically improved among low-dose irradiated lesions (mainly due to scatter dose related to anatomic proximity to another targeted lesion) compared to no-dose (<1 Gy) lesions (52, 53). Similarly, when LDI (4.9 Gy, range 2-8 Gy in 2 Gy fractions) was given intentionally to one large lesion together with ICI and HDFI, the low-dose treated lesion shrunk by 28.2% on average in 6 out of 9 patients with metastatic NSCLC (51). The RACIN trial is currently assessing the benefit of LDI to several lesions among advanced TIL-negative tumors in association with nivolumab and other agents (NCT03728179).

## Controversies About Irradiated Target Volumes

### Which Target Volumes for Ablative Irradiation When IO Is Added?

#### Tumor Irradiation

When tumoricidal irradiation of a limited disease burden (either non-metastatic or oligometastatic) is the main objective with the potential benefit of adding IO, several original approaches can then be considered to increase the therapeutic window in order to increase both the *in situ* vaccination effect and the local control while minimizing the toxicity.

One of the basic principles of RT is to ensure the full coverage of the tumor by the prescribed dose, using successive margins around the macroscopic target to account for microscopic disease (Clinical Target Volume – CTV – margins), target internal motions and patient set up (Planning Target Volume – PTV – margins). The aim is to avoid any lower dose regions which are classically associated with sites of recurrence. The correlate is the irradiation of a consequent amount of healthy tissues, with the risk of radiation-induced toxicity.

The reduction of the irradiated tissue volume would lead to the theoretical sparing of tumor-associated lymphocytes from the peri-tumoral TME, which can be rich in immune cells and can contain tertiary lymphoid structures (54). This sparing strategy could lead to a pro-immunogenic effect by sparing effector TILs which could otherwise be depleted following irradiation (54). It could also avoid the enhancement of proliferation and suppressive function of intra-tumoral T-reg, which has been shown following stereotactic irradiation (55). Such a reduction of the irradiated volume could be achieved through classical approaches of image-guided radiotherapy (IGRT) or gating/tracking strategies for mobile targets, aiming at reducing PTV margins (56). Another controversial approach would be to decrease or even to omit the Clinical Target Volume, based on the hypothesis that in the context of RT + IO, the benefit of sparing TIL would outweigh the benefit of eradicating microscopic disease. However, this hypothesis of a potential benefit from sparing peritumoral TME effector TILs arises from the notion that when large peritumoral volumes are irradiated, peritumoral TILs will mediate the local immune response as they are recruited *after* the irradiation, while those TILs present within or around the tumor at the time of irradiation, which are thought to be highly radiosensitive, are killed and cannot play an anti-tumor effector role. A recent preclinical study challenged this concept (57). The authors showed that many preexistent T cells not only survived following irradiation (yet with compromised proliferation), but also could mediate antitumor immunity *via* improvement of effector functions 9 days after irradiation as compared to T cells from unirradiated tumor (increased IFN-γ production and increased motility), without the contribution of newly infiltrating T cells. Furthermore, transcriptomic analyses suggested a T-cell reprogramming in the TME regulated by TGF-β with enriched signatures related to angiogenesis, adhesion or epithelial-mesenchymal transition, leading to a non-lymphoid tissue resident memory T-cell (T_RM_)-like phenotype. These observations are fundamental, as not all T-cell subsets are equally sensitive, with T_RM_ being more radioresistant than naïve or lymphoid tissue T cells (58, 59).

While an *in situ* vaccination effect has been shown to be crucial to achieve abscopal responses and to maximize systemic disease control, local control remains critical especially in the context of limited disease. In this perspective, partial tumor irradiation has also emerged as an innovative concept in order to widen the therapeutic window, especially for large tumors situated close to organs at risk where the classical approach of ablative RT to the whole target is challenging. While radiation oncologists usually make sure that the whole lesion receives the tumoricidal prescribed dose so that no area is underdosed, the partial irradiation approach consists in deliberately excluding a portion of the tumor from the radiation field. In two murine models, Markovsky et al. suggested that partial tumor volume irradiation (10 Gy, 15 Gy or 20 Gy delivered to 50% of the tumor using a 2 x 2 cm collimator) led to tumor responses similar to full tumor volume irradiation (10 Gy, 15 Gy or 20 Gy delivered to 100% of the tumor) *via* an immunostimulatory mechanism involving an increase in CD8^+^ T-cell traffic throughout the non-irradiated portion mediated by an increase in ICAM (60). This led to the concept of ADscopal response (61), with an immune-mediated indirect therapeutic effect of RT “close to the irradiated target” (“bystander effect”) rather than away from the target (ABscopal). Clinical data seem to corroborate this hypothesis, as large tumors (>65mL) partially irradiated exhibited local control similar to smaller fully irradiated tumors in the NRG-BR001 phase I trial of SRT (3 x 15 Gy, 5 x 10 Gy or 3 x 10 Gy) in combination with anti-PD-L1. In the partial irradiation group, mean GTV size was 177 cc, and the mean volume of GTV excluded from the irradiated target was 113 cc (19, 61). This concept should be regarded with caution, however, since the “non-irradiated” portion receives non-tumoricidal but significant doses (scatter dose) that could be sufficient to elicit an immune response. Indeed, in the study by Markovsky et al., the non-irradiated tumor sub-volume received a dose of 5% (i.e. 0.5 Gy – 1Gy) or less of the primary in-field dose, and in the NRG-BR001 trial, the median isodose line covering the original GTV in the partially irradiated group was the 13% isodose line (i.e. 3.9 Gy – 6.5 Gy in 3 to 5 fractions). Therefore, we could hypothesize that the ADscopal effect is in effect a response to LDI. The phase II PembroX trial among patients with stage I-IIIA NSCLC is currently assessing the benefit of pre-operative SRT (1 fraction of 12 Gy) to half of the primary tumor following pembrolizumab (NCT03217071). The primary endpoint for this study is the change in number of TILs in the lung cancer tissue from before and after the neo-adjuvant treatment.

Finally, it has been recently suggested that the choice of the tumor portion to be irradiated in a partial irradiation approach could be successfully guided by metabolic imaging in order to focus on the hypoxic radioresistant portion. In this perspective, Tubin et al. proposed an innovative approach of Stereotactic Body RadioTherapy targeting Partial Tumor Hypoxic (SBRT-PATHY) clonogenic cells for the treatment of bulky locally advanced NSCLC not amenable to chemo-radiotherapy, with promising rates of ADscopal and ABscopal effects of 96% and 52%, respectively (62). The hypoxic area was defined with both ^18^FDG PET-CT and contrast-enhanced CT. No CTV or PTV margin was used to limit the surrounding irradiated tissue. In this context, the accurate identification of radioresistant areas within the tumor could be of particular interest to define relevant sub-volumes to be partially irradiated. Given that hypoxia is a classical contributor to radioresistance (63) and that tumor hypoxia was shown to correlate with poor outcome in NSCLC (64), hypoxia imaging, using PET-CT with specific tracers (FMISO (flouromisonidazole), Cu-ATSM (Cu(ll)-diacetyl-bis(A/4-methylthiosemicarbazone) or FAZA = fluoroazomycin) or oxygen-enhanced MRI would help in identifying such sub-volumes (65, 66).

#### Tumor-Draining Lymph Node Irradiation

Once the estimated risk of micrometastatic spread is estimated to be high (generally over 10-15%), prophylactic irradiation of tumor-draining lymph nodes at a dose a 45-50 Gy for locally advanced disease (known as Elective Nodal Irradiation – ENI) is a classical approach in RT for several tumors such as head and neck cancers or cervical cancers. However, this practice is likely to disrupt a potential radiation-driven adaptive immune response, especially in the context of RT + IO.

In a preclinical model, Marciscano et al. showed that SRT + ENI in comparison with SRT alone restrained the adaptive immune response following SRT by modulating the chemoattractant and chemokine signature, leading to the reduction of tumor-specific effector T-cell intra-tumoral infiltration and an unfavorable balance between effector T cells and T-regs. Furthermore, ENI was shown to attenuate the combinatorial efficacy of RT and anti-CTLA-4 (67). Similar findings were recently obtained, together with the role of tumor-draining lymph nodes as a reservoir of “stem-like” anti-tumor CD8^+^ T cells, which then differentiate into terminally differentiated effectors, and the detrimental effect of RT towards lymph nodes where such cell populations are expanding (68). Finally, a major study by Dammeijer et al. suggested that, while PD-1/PD-L1 blockade therapy is generally thought to reinvigorate progenitor-exhausted T cells and to relieve tumor T-cell-mediated suppression in the TME, tumor-draining lymph nodes are a major component of anti-PD-1/PD-L1-mediated tumor immunity. Indeed, PD-L1 is also expressed by non-tumor macrophages and DCs, and the authors showed that tumor-draining lymph nodes are enriched in PD-1^+^ T cells. In addition, the selective targeting of PD-L1 only in tumor-draining lymph nodes demonstrated effective anti-tumor T-cell responses, and PD-1/PD-L1 interaction in tumor-draining lymph nodes, but not in the tumor, was correlated with prognosis in melanoma (69).

On the other hand, the omission of RT on pathologically involved lymph nodes when ENI is omitted could also be deleterious, not only because microscopic disease is not targeted but also because tumor cells confer tolerogenic features to tumor-draining lymph nodes (70).

Finally, innovative trials combining RT + IO for localized disease should be conducted to assess the benefit of omitting ENI for localized/locally advanced disease.

### Which Target Volumes for RT Added to IO?

#### High Dose per Fraction Irradiation for *In Situ* Vaccination Effect

In the polymetastatic disease setting where tumoricidal irradiation of the whole tumor burden is not feasible, the optimization of RT to be added to the IO backbone is also critical to promote the pro-immunogenic effects of RT while ensuring acceptable toxicity.

Partial irradiation has already been discussed and can be proposed in this setting to induce both abscopal and adscopal effects. The NIRVANA-Lung trial has implemented such an approach (NCT03774732).

The choice of the best tumor sites to be irradiated is also of the utmost importance, since radiation-mediated immunogenicity differs according to the target due to inherent differences in organ-related TMEs. McGee et al. prospectively monitored the peripheral immune response following SRT to any organ. They found that SRT to parenchymal sites (liver or lung) but not to bone or brain induced changes in systemic immunophenotypes, including a decrease in total and cytotoxic NK cells, an increase in TIM3^+^ NK cells and activated memory CD4^+^ and CD8^+^ T cells, and a decrease in circulating levels of chemoattractant chemokines (71). This differential pattern can thus be explained by differences in antigenic load and relative abundance of innate immune cells and lymphocytes between these organs. However, one cannot rule out the impact of different dose/fractionation schedules between parenchymal lesions versus bone/brain lesions in that study. Moreover, in their phase I trial testing SRT + ipilimumab for metastatic tumors within lung or liver, a team from MD Anderson estimated that patients having received SRT to the liver as compared to the lung presented a transient increase in markers suggestive of enhanced peripheral T-cell activation, i.e. higher proportions of CD8^+^ T cells expressing ICOS, GITR, and 4-1BB (72). However, this increased peripheral immune activation following SRT did not translate into clinical responses, as in the phase II trial assessing the same combination of SRT + ipilimumab, the same group found that the rates of clinical benefit of non-irradiated tumor volume were 31% for irradiated lung versus 14% for irradiated liver metastases (P=0.061) (53). This discordance could be due to the inherent adverse prognosis of liver metastases, but also to a lack of concordance between peripheral immune correlates and intra-tumoral immunologic patterns. More recently, Yu et al. observed that the presence of liver metastases negatively correlates with response to IO among patients with melanoma and NSCLC, independently of other established biomarkers of response, and that liver metastases, but not lung metastases, modulate immune function in animal models and in patients by reducing the number and the function of peripheral antigen-specific T cells. In-depth analysis revealed that hepatic CD11b^+^F4/80^+^ monocyte-derived macrophages can induce antigen-specific CD8^+^ T cell apoptosis *via* the Fas-FasL pathway in the liver metastatic TME, suggesting that liver metastases siphon and eliminate antigen-specific CD8^+^ T cells, creating a systemic immune desert in preclinical models. Interestingly, liver metastasis-directed RT in preclinical models was able to reshape the liver TME by eliminating immunosuppressive hepatic macrophages, thereby preventing antigen-specific T cell loss (73). These data provide a new synergistic explanation of how the association of RT and IO improves the efficacy of IO, and make liver metastases key tumor sites to be irradiated to promote systemic antitumor immunity.

Finally, the classical approach to induce the abscopal effect in the polymetastatic setting in association with IO is a single-site irradiation approach. It has not yielded strong evidence as two phase II trials in NSCLC and head and neck cancers failed to meet their objective of out-of-field overall response rate (22, 74). More recently, a multifactorial rationale has emerged to target as many lesions as possible in this context (75). First, the cytoreductive effect of multi-target irradiation potentiates the destruction of resistant subclonal populations. Second, due to differences in immunogenicity owing to distinct TME features between organs (71), a multitarget approach, preferentially in different organs, would potentiate the *in situ* vaccination effect. Furthermore, considering tumor heterogeneity, the release of a wide variety of distinct TAs would intuitively increase the chance of successful priming of anti-TA T cells and the constitution of a wide clonal T-cell repertoire, leading to an efficient CD8^+^-mediated cytotoxic effect towards shared TA in distinct lesions. Formenti et al. thus suggested that the expansion of a large number of tumor-specific T-cell clones in peripheral blood correlates well with the achievement of abscopal responses in NSCLC patients treated with SRT + ipilimumab (20). Additionally, the irradiation of multiple sites could optimize the recruitment and the homing of immune cells through modification in microvasculature and the secretion of chemoattractant chemokines in those sites (8, 9). Finally, it has been shown that exhausted T cells arise from effector T cells, which gradually lose their effector functions and express multiple inhibitory receptors due to continuous T-cell receptor (TCR) stimulation from persistent antigen exposure, either in the context of chronic infections or of cancer (76). Therefore, a high tumor burden can be regarded as a source of persistent antigen exposure, so the maximal reduction in tumor burden through multi-target HDFI would lead to a decrease in T-cell exhaustion. This hypothesis has been suggested by Huang et al. who demonstrated that among patients treated with the anti-PD-1 agent pembrolizumab, the clinical benefit was strongly correlated with the magnitude of reinvigoration of exhausted CD8^+^ T cells (as indicated by Ki67 expression and IFN-γ production), but above all with the amount of initial tumor burden, with greater tumor burden resulting in lower response rates. This led to the concept of a “reinvigoration-to-tumor burden” ratio as a positive predictive factor of response to checkpoint inhibitors when the ratio is high (77). These results are in line with several clinical reports revealing an increased benefit of ICI among patients with polymetastatic melanoma with lower tumor burden (78, 79), and major benefits of ICI in non-metastatic situations with a high risk of micrometastases (2, 80). Additionally, in a subgroup analysis from a randomized phase III trial comparing RT to 1-5 bone lesions (single dose of 8Gy) to the same RT + ipilimumab among patients with castration-resistant prostate cancer, the improvement in OS with the addition of ipilimumab favored those patients with fewer lesions (23).

Overall, several clinical trials have reported the results of multi-target SRT in association with IO in a polymetastatic setting (19, 81) and have yielded mixed results. Luke et al. reported the results of a phase I trial assessing SRT to 2-4 lesions (majority with 2 sites treated) followed by pembrolizumab in patients with heavily pretreated metastatic solid tumors. The overall response rate was 13%, and was similar to that from historical series of pembrolizumab alone (19). In the phase I/II from Welsh et al. among NSCLC patients, the best out-of-field response was similar between pembrolizumab alone and pembrolizumab + SRT to one to four lesions (81). However, the multi-target approach with irradiation of as many targets as possible should probably be preferred in the future. The phase III NIRVANA-Lung trial has implemented such an approach in its design.

In the context of oligometastatic disease, the added value of adjoining SRT to the whole tumor burden to IO is supported by a rationale which goes beyond the pure benefit of exclusive ablative RT suggested in several trials (38, 39, 82, 83). This rationale has been already partly discussed and includes the following: the optimization of the systemic response against subclinical disease through a multitarget strategy (and optimization of *in situ* vaccination) and of the reinvigoration-to-tumor burden ratio *via* a complete cytoreductive effect (77); the optimization of the local antitumor immune response from the preexistent TILs (57); and the frequent failure in sites of initial disease under IO (84, 85). Accordingly, Bauml et al. performed a single-arm phase II trial in 45 patients with oligometastatic NSCLC (≤ 4 sites) who were treated by local ablative therapy followed by pembrolizumab (86). The median PFS was as high as 19.1 months (versus 6.6 months in historical series) and the 2-year OS was remarkably high (77.5%).

#### Which Volume for Low-Dose Irradiation?

As previously discussed, LDI is able to reprogram the TME leading to T-cell homing towards tumor sites, and original approaches combining high-dose RT, low-dose irradiation and IO (triple therapy), are gaining evidence (51). Yet, the question of the number of lesions to be treated with LDI remains unanswered.

In a pragmatic approach, LDI could be performed for lesions not suitable for HDFI or with higher risk of toxicity, such as large lesions (more than 5 cm) or lesions near critical organs at risk, for example ultra-central lung lesions abutting the proximal bronchial tree (87, 88).

More provocatively, LDI could be delivered to large volumes such as whole-abdominal irradiation or even whole-body irradiation, aiming at targeting all tumor lesions. Several preclinical studies support this hypothesis (89, 90). Recently, Liu et al. used a combination of HDFI (8 Gy x 3) with low-dose total body irradiation (0.1 Gy) in syngeneic mouse models of breast and colon carcinoma and found an enhanced systemic anti-tumor response as compared to HDFI alone, by infiltration of CD8^+^ T cells dependent on IFN-γ and alteration of the immunosuppressive TME of secondary tumors (89).

The concern of late toxicity from large volume irradiation remains, even with LDI, especially regarding myelosuppression or toxicity related to lung, liver or kidney injury (91). A promising alternative would be to irradiate the whole macroscopic lesions using intensity modulation radiotherapy (IMRT) techniques, while sparing bone marrow and any non-target organ.

## New Concepts for Dose to Organs at Risk: Dose to Immune Organs at Risk (iOAR)

### Impact of RT on Lymphocytes

Radiation-induced lymphopenia (RILP) has been known for decades and has been extensively described since then (92–95). It partly explains the immune suppressive effects following RT. Lymphocytes are the most radiosensitive cells within the body due to prominent apoptotic response pathways. Lethal doses to reduce the surviving fraction of circulating CD4^+^ and CD8^+^ T lymphocytes by 90% (DL90), 50% (DL50) and 10% (DL10) are only 3Gy, 2 Gy and 0.5Gy, respectively (96).

The mechanisms of RILP involve irradiation of circulating lymphocytes as well as lymphocyte-rich areas in lymphoid organs or, potentially, within the tumor (54). For example, patients who receive prophylactic lymph node irradiation experience more frequent and more profound RILP than those who do not receive it (97–99). The same observation has been made for patients with abdominal tumors who undergo irradiation of large splenic volumes (100). However, not all T-cell subsets are equally radiosensitive, with regulatory, activated and memory T cells having been shown to be more resistant than naive T cells, and with non-lymphoid T_RM_ and intra-tumoral T-cells being more resistant than lymphoid tissue and circulating T cells (57–59, 101–103). Overall, RT mainly induces a decrease in naïve T cells, which drives a decrease in absolute lymphocyte count and an enrichment in T-regs, with no disruption of the functionality of T lymphocytes or the frequency of antigen-specific CD8^+^ T cells (104, 105).

### Impact of Lymphopenia on Outcome

Classically, RILP affects more than half of patients receiving RT and is transient with a recovery mostly within 3-6 months after RT, but with prolonged depletion in some cases (16, 106). The adjunction of concurrent chemotherapy can increase the severity of RILP (107). The impact of RILP has been extensively explored in several tumor types (16). In the context of NSCLC, several studies have demonstrated the negative impact of RILP on OS and PFS (43, 108–110). Additionally, baseline lymphopenia has been negatively correlated with outcome following ICI for the treatment of solid tumors (111–113). Therefore, in the context of the RT-IO combination, attention should be paid to limiting the severity of RILP. A recent retrospective series suggested that among patients treated by ICI for metastatic tumors, RILP following palliative RT at onset of ICI therapy was associated with poorer outcome (114).

### Factors Predicting RILP

Apart from patient-related factors such as advanced age, smoking habits, comedications, baseline lymphopenia or even genetic factors (115), several factors related to the characteristics of RT have been associated with the incidence and severity of RILP. These factors are directly or indirectly correlated with the amount of circulating lymphocytes and lymphoid organs exposed to (even low) doses of radiation, and with the duration of exposure. Thus, considering blood flow, any factor leading to prolonged RT duration will increase the amount of blood passing through the beam, and could potentially increase the severity of RILP. For example, an increased number of fractions (through hyperfractionation with twice-daily fractions) has been shown to be a risk factor for RILP (43, 116, 117). Similarly, a low-dose rate should be avoided intuitively, although evidence is lacking.

Furthermore, irradiation of organs containing large blood volumes and/or with high blood flow velocity could be at risk of RILP. Recently, among 244 patients treated by chemo-radiotherapy for NSCLC, the heart volume receiving 20 Gy or more (V20Gy) and 40 Gy or more (V40Gy) was significantly correlated with the 1-month post-RT start neutrophil-to-lymphocyte ratio (NLR) (118). Similarly, Contreras et al. found that among patients with NSCLC treated with definitive RT (± chemotherapy), a heart V50Gy > 25% was significantly associated with a higher NLR 4 months post-RT (119). Similarly, lung V5Gy was significantly and independently associated with post-RT lymphocyte nadir among 711 patients who received definitive RT for NSCLC (43). Abravan et al. found that mean heart dose and mean lung dose were correlated, and that a thoracic vertebrae V20Gy was correlated with grade ≥3 lymphopenia following thoracic RT (110). However, in a recent analysis, Joseph et al. did not find any correlation between heart or lung dosimetric parameters and severity of RILP in multivariate analysis, but rather demonstrated a negative correlation between integral body dose and post-treatment absolute lymphocyte count, suggesting the detrimental effect of a “low-dose bath” (108).

The amount of circulating lymphocytes exposed to radiation dose also seems to be correlated with the size of the gross tumor volume to be irradiated. For example, larger GTV were associated with lower lymphocyte nadir among patients treated for NSCLC (43) or glioblastoma (120). Similarly, the amount of spleen exposed to low/medium doses (V5Gy, V10Gy, V15Gy, V20Gy, mean dose) has been correlated with severe post-chemoradiotherapy lymphopenia in patients treated for locally advanced pancreatic cancer (100, 121).

Finally, several models have been proposed to estimate the dose delivered to circulating immune cells. Yovino et al. established an *in silico* model to estimate the radiation dose to circulating lymphocytes during a standard radiation treatment of 60 Gy in 30 fractions for glioblastoma. The model indicated that while a single fraction of 2 Gy delivered ≥ 0.5 Gy to 5% of the total blood pool, 99% of circulating cells had received ≥ 0.5 Gy after 30 fractions (120). Similarly, Jin et al. developed a three-step model to calculate the effective dose to the immune cells (EDIC) during thoracic RT, assuming the following: a) the dose to circulating immune cells including rapidly circulating ones in the heart, lung and blood vessels, and slowly circulating ones in the lymphatic system and blood reservoirs (a portion of veins/capillaries) is a surrogate for the EDIC; b) at each fraction, the radiation dose is uniformly delivered to all cells for rapidly circulating ones, and only to those in the irradiated volume for slowly circulating cells. In this model, the blood dose relating to the contribution of a given organ is approximated by its mean organ dose (MOD), the percentage of cardiac output, the percentage of blood volume it receives, the time for one blood circulation, the irradiation time and the number of fractions (120). Second, the EUD (Equivalent Uniform Dose) is determined from a blood dose/volume histogram (percentage of blood volume irradiated at a given dose). Third, the EDIC is the sum of the EUDs of each organ. In summary, the EDIC can be approximated as a function of the mean heart dose, the mean lung dose, the mean body dose and the number of fractions (122). Using this model, Ladbury et al. showed that among 117 patients with stage III NSCLC treated with definitive fractionated radiation, most of whom were receiving concurrent chemotherapy, a higher EDIC was correlated with a greater risk of grade ≥ 3 lymphopenia (123). Corroborating the impact of tumor volume on severity of RILP, they also found that the planning target volume (PTV) was strongly associated with the EDIC with a 1.7 Gy increase per liter (p < 0.05).

### Optimizing Dose to iOAR

To limit the impact of radiation dose to the host immune system, one can hypothesize that the limitation of radiation dose to circulating lymphocytes as well as to lymphocyte-rich areas in lymphoid organs could be beneficial. To do so, and especially for thoracic malignancies, RT planning should be performed in such a way that doses to relevant organs or structures are as low as possible. These organs include the following: heart (possible impact of V20Gy, V40Gy, V50Gy, mean dose) (110, 118, 119), lung (possible impact of V5Gy, mean dose) (43, 110), large vessels, non-involved draining lymph nodes, bone-marrow within spine (possible impact of V20Gy) (110) or pelvis mostly, spleen (possible impact of V5Gy, V10Gy, V15Gy, V20Gy and mean dose) (100, 121), gut and thymus in children.

Interestingly, by applying the global concept of EDIC to estimate the dose to the immune system as an OAR rather than focusing on separate OAR involved in the process of RILP, a secondary analysis of the RTOG 0617 trial found that EDIC was the strongest significant factor for OS, PFS and local PFS (LPFS) in multivariate analysis following chemo-radiotherapy for stage III NSCLC, with a high EDIC associated with worse outcome. While GTV, mean heart dose, mean lung dose and integral dose were significant factors in a multivariate model without EDIC, they were no longer significant when EDIC was added (122). These findings were validated externally by Ladbury et al. In their series of stage III NSCLC treated with radical RT, they found that EDIC was an independent factor for OS, LPFS and PFS. Furthermore, plotting OS and LPFS hazard ratios as a function of EDIC suggested that the most profound effect on OS and LPFS occurred when EDIC was above 6.3Gy (123). Similarly, EDIC was also an independent factor of OS among 92 patients with esophageal squamous cell carcinoma treated with neoadjuvant chemo-radiotherapy (124). However, since the EDIC model is a measure of radiation dose to circulating immune cells, this correlation could be confounded by other organs at risk or structures such as the spleen, bone marrow and lymph nodes. Furthermore, the model does not account for interplay between radiation and chemotherapy.

Finally, further investigation is needed to optimize the dose to immune-related OARs with defined thresholds, especially in the context of RT-IO combinations.

## Insights From Dose Delivery

The modality of delivery of the radiation dose should be taken into account when investigating the immune effects of RT.

Intensity-modulated radiotherapy (IMRT) emerged in clinical practice around two decades ago as a technique for delivering a more accurate dose distribution than conventional 2-dimensional RT or 3-dimensional conformal RT (3D-CRT), with limited exposure of adjacent OARs, including structures located within a concave area of the PTV. To do so, an “inverse planning” is performed, where the treatment planner first determines the dose distribution for the target tumor and OARs, and then the optimization method determines the intensity of the irradiation beam (125). In stage III NSCLC, IMRT as compared to 3D-CRT has been associated in dosimetric studies with improved PTV coverage, and with a decrease in the volume of whole lung receiving more than 20 Gy and in cardiac doses (126). This translated into a reduction in severe pulmonary toxicity and even improved OS in several large retrospective series (127–130). IMRT was also associated with decreased severe radiation pneumonitis as well as improved quality of life in secondary analyses of the RTOG0617 trial (131, 132). However, in comparison to 3D-CRT, IMRT increases the low-dose bath as a greater number of beams and monitor units are used, and several studies have shown an increase in lung V5Gy (126, 133). Concerns about V5Gy/V10Gy and fatal pneumonitis have been raised with IMRT in the context of post-operative RT for mesothelioma (134), however, no clear correlation has been established for IMRT in NSCLC, and it is commonly thought that the potential benefit of IMRT outweighs this risk in NSCLC. Nevertheless, as lung V5Gy was significantly associated with post-RT lymphocyte nadir among patients who received definitive RT for NSCLC (43), and given the negative impact of lymphopenia on outcome, attention should be paid to the low-dose radiation lung volume in the era of immunotherapy. This potential increased risk of lymphopenia with IMRT could be counterbalanced by a decrease in treatment time (beam on time) by using flattening filter free (FFF) radiation beams, which can provide high-dose rate beams (135).

Stereotactic RT is usually proposed for early-stage NSCLC in medically inoperable patients. Owing to the technical properties and characteristics of dose gradient, SRT is associated with low-dose bath to ensure a high conformal dose distribution around the target; however considering the small size of the lesions treated with SRT, this low-dose spread is usually limited. This could prompt to develop approaches of SRT-based sub-volume radiation boost following a conventionally fractionated course of RT for the treatment of locally advanced disease; this approach is currently being explored in stage III NSCLC (136).

Proton therapy is also gaining interest in locally advanced NSCLC owing to its dose distribution capabilities related to the release of proton energy, mostly at the end of the path, a phenomenon known as the Bragg peak phenomenon. Proton therapy has shown promise in reducing normal lung tissues receiving low-dose ranges, while maintaining dose constraints to other critical structures such as the heart, esophagus and spinal cord (137). This could explain the superiority of proton therapy over photon-based IMRT in terms of severe lymphopenia in patients treated with (chemo-)radiotherapy for glioblastoma (138), esophageal cancer (139, 140) or medulloblastoma (141). However, a phase II randomized trial comparing proton therapy and IMRT in the treatment of stage III NSCLC failed to show any advantage of proton therapy on toxicity or on local failures (142). In addition to this dosimetric advantage, proton therapy could have intrinsic immunomodulatory properties. A recent study suggests that proton therapy induces upregulation of surface molecules involved in immune recognition (HLA, ICAM-1 and tumor associated antigens), and translocation of calreticulin, in a manner similar to photon irradiation. The authors extended their observations to cancer stem cells, which are classically resistant to radiation (143, 144). These results also support the association of proton therapy with T-cell mediated immunotherapy.

Finally, owing to their particular features, emerging unconventional approaches of RT may provide additional benefits when combined with IO (145). FLASH RT is one of these promising approaches. It is able to deliver radiation at ultra-high dose rate, which is thought to induce massive oxygen consumption; while tumors are generally already hypoxic, FLASH RT can induce transient protective hypoxia in normal tissues. Therefore, FLASH RT could enhance the differential effect between tumors and normal tissues as compared to conventional RT (146). The modulation of immune response with FLASH RT is not well established; however, together with a decrease in treatment time, some particular features associated with FLASH RT such as massive TA release or decrease in immunosuppressive TGF-β cascade activation may provide additional mechanisms of the synergistic effect of the RT – IO association (147, 148).

## Discussion

**Figure 2:**
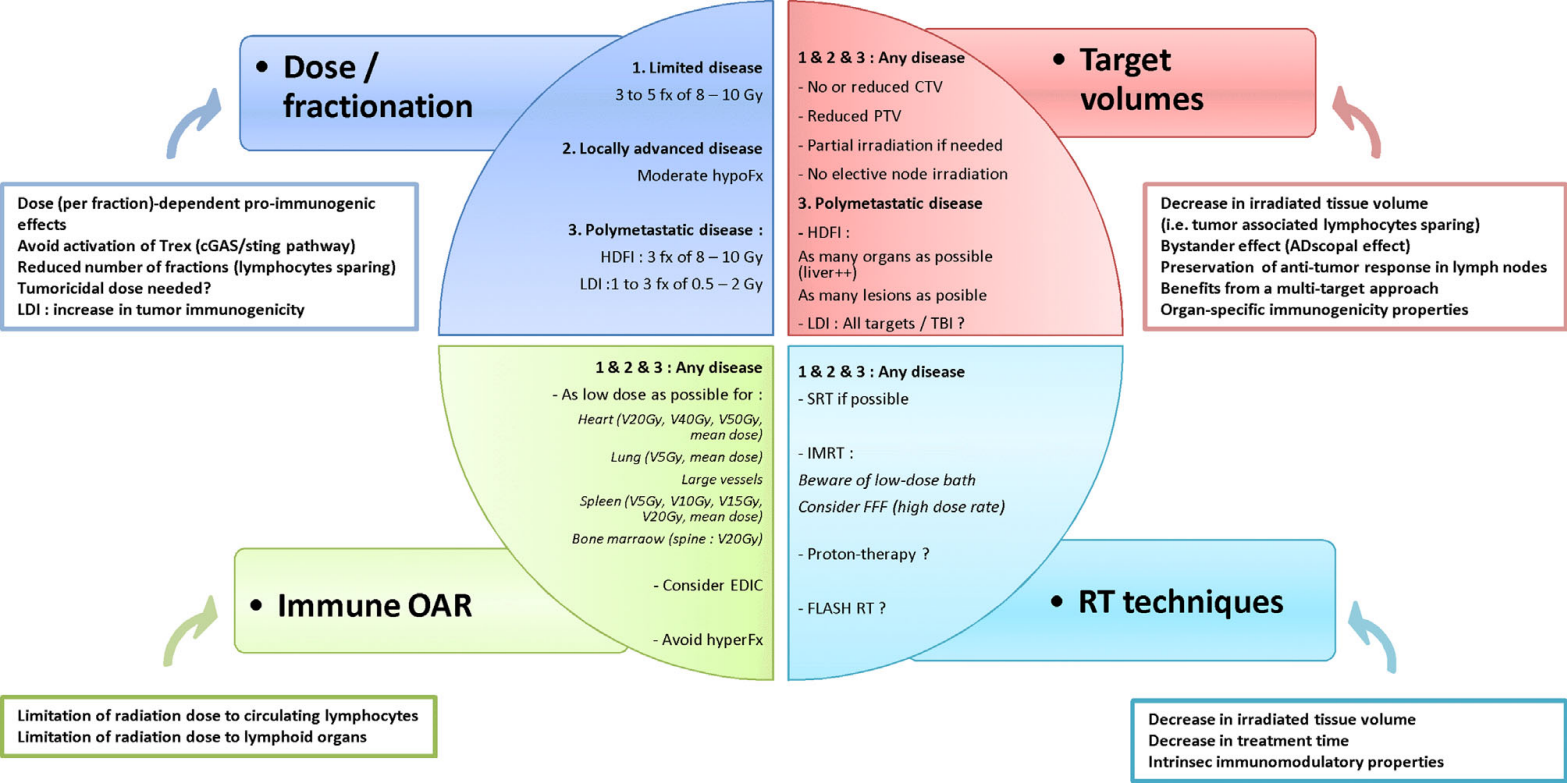
Hypothesis of framework to optimize radiotherapy-immunotherapy combination. fx, fraction; hypoFx/hyperFx, hypofractionation/hyperfractionation; CTV, clinical target volume; PTV, planning target volume; HDFI, high-dose per fraction irradiation; LDI, low-dose irradiation; OAR, organs at risk; VxGy, volume of organ receiving at least x Gy; EDIC, Effective Dose to Immune Cells; SRT, stereotactic radiotherapy; IMRT, intensity-modulated radiotherapy; FFF, Flattening Filter Free.

The combination of RT and IO at any stage of cancer disease, i.e. from early stage to both oligo- and poly-metastatic disease, is offering new hope for the treatment of patients with malignancies. However, given the dual effect of RT upon the host immune system the RT schedule must be optimized whenever a synergistic effect of the combination of RT and IO is expected. To reach this objective, several traditional dogmas about RT might need to be revisited and challenged in this new therapeutic era, regarding dose, fractionation, target volumes, dose to organs at risk and dose delivery techniques. The main issues are summarized in **Figure 2**. Thus, both translational and clinical studies are necessary to better understand the mechanisms underlying the immune effects of RT and to provide a strong rationale for this combination. Along with the optimization of radiation dose delivery, biomarkers need to be validated to predict a synergistic effect of the RT – IO combination, based upon tissue analysis, circulating biomarkers, and quantitative imaging with radiomics (149, 150).

## Author Contributions

JK designed the review, analyzed the data and drafted the manuscript. JM, CG-R, EC-J and MA analyzed the data and critically revised the manuscript. EC-J and MA supervised the writing. All authors contributed to the article and approved the submitted version.

## Funding

Open access publication fees funded by Institut Claudius Regaud/Institut Universitaire du Cancer de Toulouse - Oncopole.

## Conflict of Interest

The authors declare that the research was conducted in the absence of any commercial or financial relationships that could be construed as a potential conflict of interest.
